# Efficacy of Step-Down Therapy Using Vonoprazan for Symptomatic Mild Reflux Esophagitis

**DOI:** 10.1155/grp/5620034

**Published:** 2024-11-14

**Authors:** Yorinari Ochiai, Daisuke Kikuchi, Shu Hoteya

**Affiliations:** ^1^Department of Gastroenterology, Toranomon Hospital, Tokyo, Japan; ^2^Department of Gastroenterology, Toranomon Hospital Kajigaya, Kawasaki, Kanagawa, Japan

## Abstract

**Objective:** Patient-reported outcomes (PROs) are becoming pivotal in managing gastroesophageal reflux disease (GERD). Current Japanese guidelines for GERD recommend vonoprazan (VPZ) as a treatment option for mild reflux esophagitis (RE). However, it has been hypothesized that 4 weeks of VPZ 20 mg is not always necessary for mild RE if the treatment outcome is based on patient symptoms. This study is aimed at elucidating the efficacy of a new tapering therapeutic strategy (step-down therapy) using VPZ for symptomatic mild RE based on PRO.

**Methods:** This multicenter retrospective study examined VPZ's efficacy for step-down therapy between October 2021 and November 2022. Included were 63 consecutive patients from the outpatient clinics of Toranomon Hospital and Toranomon Hospital Kajigaya with symptoms associated with RE (frequency scale for the symptoms of GERD (FSSG) scores ≥ 8 points) and mild RE classified as the Los Angeles Classification Grade A or B. Step-down therapy was defined as a treatment period of 28 days. VPZ 20 mg was administered as the initial treatment. Afterward, patients were allowed to step down to VPZ 10 mg if their symptoms improved, and VPZ could be discontinued if symptoms disappeared. If symptoms worsened, the dosage could be increased from 10 to 20 mg VPZ, or VPZ could be restarted at 20 mg after discontinuation.

**Results:** The proportion of patients whose FSSG scores decreased by ≥ 3 points with step-down therapy was 76.2% (48/63 patients). The median FSSG scores before and after step-down therapy were 13 (range, 8–35) and 7 (range, 0–29), respectively (*p* < 0.01). Overall, 71.4% (45/63) of the patients stepped down from 20 to 10 mg VPZ, and 46% (29/63) of the patients discontinued VPZ.

**Conclusion:** Step-down therapy using VPZ may be a viable treatment option for symptomatic patients with mild RE.

## 1. Introduction

The prevalence of gastroesophageal reflux disease (GERD) in Japan, now a major health concern, began to increase during the late 1990s [1]. The reported occurrences of erosive reflux disease and nonerosive reflux disease among Japanese patients with GERD are 41.4% and 58.6%, respectively [1]. According to the Los Angeles (LA) classification, the severity grades are as follows: Grade A, 54.6%; Grade B, 32.4%; and Grade C + D, 13% in Japan [1].

Vonoprazan (VPZ), a novel potassium-competitive acid blocker that blocks the H^+^/K^+^ ATPase competitively and reversibly [2], has been available in Japan since 2015. VPZ allows rapid, profound, and sustained suppression of gastric acid secretion with a single oral dose, regardless of *CYP2C19* polymorphisms, and is well tolerated by healthy male patients [3]. Previous reports indicate that VPZ 20 mg has more rapid and sustained acid-inhibitory effects than esomeprazole 20 mg or rabeprazole 10 mg [4]. Additionally, a systematic review showed that the GERD healing effect of VPZ was higher than that of rabeprazole 20 mg. In the study, VPZ was more effective than most proton pump inhibitors (PPIs) for patients with severe erosive esophagitis [5].

Evidence-based clinical practice guidelines for GERD were published in 2021 (revised Japanese third edition) [6]. These guidelines clarified treatment algorithms based on the severity of reflux esophagitis (RE) and the inclusion of VPZ for GERD treatment. RE is subdivided into severe RE (Grade C or D according to the LA classification) and mild RE (Grade A or B according to the LA classification) [6]. Therapy for severe RE includes VPZ 20 mg for 4 weeks as the initial treatment and VPZ 10 mg as maintenance treatment. In contrast, a standard dose of a PPI for 8 weeks or VPZ 20 mg for 4 weeks as the initial treatment is recommended for mild RE. These recommendations prioritize endoscopic mucosal healing as the primary treatment outcome. However, previous study reported that most patients with mild RE did not progress to severe RE in the natural course [7]. Therefore, especially in mild RE cases, there is increasing recognition of the importance of symptom assessment using patient-reported outcomes (PROs) in GERD management [8, 9].

In mild RE, the median frequency scale for the symptoms of GERD (FSSG) scores improved from 13 (range 5–39) to 4 (range 0–25) after 4 weeks of treatment with VPZ 20 mg [10]. It has been hypothesized that 4 weeks of VPZ 20 mg is not always necessary for mild RE if the treatment outcome is based on the symptoms of patients. Additionally, there is a lack of evidence on the effectiveness of a new tapering therapeutic strategy for mild RE. Furthermore, tapering therapy could lead to a dose reduction of the acid suppressants. This study is aimed at elucidating the efficacy of tapering (step-down) therapy using VPZ for symptomatic mild RE.

## 2. Materials and Methods

### 2.1. Patients and Study Design

**Figure 1:**
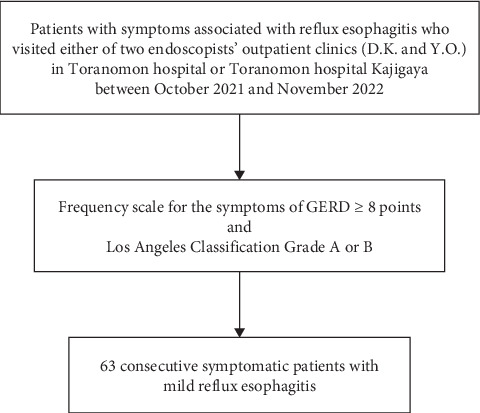
Flowchart of patient selection. GERD, gastroesophageal reflux disease.

This multicenter retrospective study included data from patients who visited the outpatient clinics of Toranomon Hospital and Toranomon Hospital Kajigaya of either of two endoscopists (D.K. and Y.O.) because of symptoms associated with RE and underwent esophagogastroduodenoscopy between October 2021 and November 2022. Among patients with RE, the inclusion criteria were an FSSG score ≥ 8 and mild RE classified as LA Grade A or B. The exclusion criteria were an FSSG score < 7 or severe RE classified as LA Grade C or D. Consequently, 63 consecutive symptomatic patients with mild RE were enrolled in this study (see Figure 1).

### 2.2. Endoscopic Evaluation

Esophagogastroduodenoscopy was performed via endoscopy (GIF-H290 or GIF-H290Z; Olympus Co., Tokyo, Japan) under full consciousness or conscious sedation, based on the patient's preference. The endoscopic severity of RE was assessed based on the LA classification [11]. LA grades were based on the judgment of the endoscopist.

### 2.3. Evaluation of Symptoms

RE symptoms were recorded using the questionnaire method and assessed using the FSSG [12, 13] before and after step-down therapy. A score of 8 was set as the cut-off value.

### 2.4. Definition of Step-Down Therapy

Step-down therapy was defined as a treatment period of 28 days. VPZ 20 mg was administered as the initial treatment. The degree of disability of daily life due to discomfort of GERD (*D* score) was used as a new GERD symptom evaluation method (see Table 1). This scoring system focuses on the impact of GERD symptoms on daily life in a simplified manner. The *D* score of “0” indicates that GERD symptoms do not interfere with daily life, while scores of “1, 2, and 3” are assigned based on how severe GERD symptoms interfere with daily life.

Patients were advised to step down VPZ if the *D* score was 0. Additionally, if the *D* score was decreasing, then patients could also opt to step down VPZ. Based on the *D* score, the decision to step down VPZ was made at the patient's discretion. For example, patients were allowed to step down to VPZ 10 mg if their symptoms improved and the *D* score decreased. After stepping down, patients could discontinue VPZ 10 mg if their symptoms disappeared and their *D* score reached 0. When symptoms worsened with an increase in the *D* score, patients could step up from VPZ 10 mg to VPZ 20 mg or restart VPZ 20 mg.

### 2.5. Primary Endpoint and Other Outcomes

**Figure 2:**
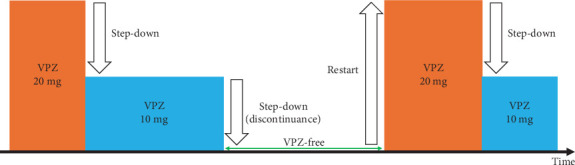
Clinical course of step-down therapy using VPZ. VPZ, vonoprazan.

The primary endpoint was the proportion of patients whose FSSG scores decreased by ≥ 3 points with step-down therapy. The proportion of patients whose FSSG scores decreased by ≥ 1 point and the proportion of patients whose FSSG scores decreased to ≤ 7 points were also evaluated. The FSSG scores before and after step-down therapy and those for heartburn before and after step-down therapy were compared. The secondary endpoints were the proportions of patients who stepped down VPZ, could not step down VPZ, discontinued VPZ, and restarted VPZ (Figure 2). We assessed the clinical course of step-down therapy during a treatment period of 28 days in a medical interview. Moreover, subgroup analyses of the LA grades and PPI use before step-down therapy were conducted.

### 2.6. Statistical Analysis

The Wilcoxon signed-rank test assessed differences in FSSG scores before and after treatment. All statistical analyses were performed using SPSS software for Windows (Version 25.0; IBM Corp., Armonk, New York, United States). Statistical significance was considered when *p* < 0.05.

### 2.7. Ethics Statement

This study was conducted in accordance with the ethical principles of the Declaration of Helsinki for medical research involving human participants. The study protocol was approved by the Ethics Committee of Toranomon Hospital in Japan (Protocol Number 2353). The requirement for informed consent was waived because the data were anonymized. The information disclosure document of this study was published on our hospital's website.

## 3. Results

### 3.1. Patient Characteristics

Table 2 lists patient characteristics. Endoscopic LA grades before step-down therapy were Grade A for 37 (58.7%) patients and Grade B for 26 (41.3%) patients. The proportion of patients who used PPI before the step-down therapy was 33.3%.

### 3.2. Main Outcomes

The primary endpoint and other secondary outcomes are listed in Table 3. The median FSSG scores before and after step-down therapy were 13 (8–35) and 7 (0–29), respectively (*p* < 0.01). The FSSG scores for heartburn before and after step-down therapy were 2 (0–4) and 1 (0–3), respectively (*p* < 0.01). The intake status of VPZ during step-down therapy and the median duration of VPZ intake according to the dose, which were secondary endpoints, are listed in Table 4.

### 3.3. Subgroup Analyses

The results of the subgroup analyses are listed in Table 5. The median FSSG scores before and after step-down therapy were significantly different in LA Grade A (*n* = 37, *p* < 0.01), LA Grade B (*n* = 26, *p* < 0.01), PPI-naïve (*n* = 42, *p* < 0.01), and PPI-use (*n* = 21, *p* = 0.01) groups.

## 4. Discussion

This study demonstrates the efficacy of step-down therapy using VPZ for symptomatic mild RE. The proportion of patients whose FSSG scores decreased by ≥ 3 points after step-down therapy was 76.2% (48/63 patients). Additionally, the median FSSG scores before and after step-down therapy were 13 (8–35) and 7 (0–29), respectively, which were significantly different (*p* < 0.01). Moreover, the FSSG scores for heartburn before and after step-down therapy were 2 (0–4) and 1 (0–3), respectively, which were also significantly different (*p* < 0.01).

Most erosive esophagitis cases in Japan are mild, such as Grade A or B, and account for 87% of all cases [1]. In Japan, the frequency of asymptomatic RE (FSSG = 0) was 11.6%, while that of symptomatic RE was 88.4% [14]. The distribution of asymptomatic RE grades was as follows: Grade A, 80.0%; Grade B, 17.8%; Grade C, 2.2%; and Grade D, 0%. Similarly, the distribution of the symptomatic RE (FSSG score ≥ 1) grades was as follows: Grade A, 72.6%; Grade B, 24.8%; Grade C, 2.0%; and Grade D, 0.6%. The differences between these two groups were not significant [14]. Thus, endoscopically severity of esophageal mucosal breaks is not necessarily correlated with subjective symptoms [15]. According to the current guidelines for GERD in Japan [6], the dosage and duration of VPZ treatment were not based on symptoms; instead, they were based on endoscopic mucosal healing. Recently, the importance of PRO in GERD treatment has been emphasized. The factor contributing most to treatment satisfaction was symptom alleviation in patients with RE receiving pharmacological therapy in actual clinical settings in Japan [16]. Few studies have focused on patient symptoms and PRO of GERD treatment with VPZ. One prospective study that enrolled patients regardless of their FSSG score reported that patients with mild RE taking VPZ 20 mg for 4 weeks experienced improvements in their FSSG scores [10]. In contrast, the present study enrolled patients with FSSG scores ≥ 8. The extent of the decrease in FSSG scores was comparable between these two studies. The median FSSG scores in the previous study were 13 and 4 before and after treatment, respectively. The median FSSG scores in the present study were 13 and 7 before and after treatment, respectively.

Both PPIs and VPZ are widely used to treat GERD; however, several concerns regarding their long-term administration have been raised [17]. Nevertheless, the quality of evidence regarding PPI-related adverse events during these observational, crossover, or randomized controlled trials was low because of possible effects of residual confounders, consistency issues, and differences in findings between observational studies and randomized controlled trials [18]. According to the current Japanese guidelines, long-term PPI therapy is generally safe; however, careful observation is required [6]. Regarding PPI-related endoscopic gastric mucosal changes, fundic gland polyps, hyperplastic polyps, multiple white and flat elevated lesions, cobblestone-like mucosa, and black spots have been reported [19, 20]. The safety of the long-term administration of VPZ remains unknown.

Regarding endoscopic findings, some case reports have described gastric polyp regression after VPZ cessation [21–23]. Dose reduction and cessation of PPIs and VPZ have been estimated to decrease side effects related to acid suppressants. Attention should be focused on these issues, especially during long-term maintenance therapy. Optimization of acid suppressant administration for those who need it is essential. Recently, VPZ-first treatment (top–down therapy) has been reported to be a cost-effective therapeutic strategy for symptomatic GERD [24]. Step-down therapy using VPZ is also a VPZ-first treatment. However, this therapeutic strategy has a feature that focuses more on the dose reduction and cessation of VPZ based on PRO.

Several advantages are associated with step-down therapy using VPZ. We consider that stepping down VPZ would be effective in alleviating rebound acid hypersecretion after cessation of VPZ. Some studies have quantified acid hypersecretion after PPI cessation due to an increase in gastric acid secretion capacity caused by hypergastrinemia [25]. It was reported that the increase in serum gastrin level was greater in VPZ 20 mg than in VPZ 10 mg [26]. Additionally, VPZ has been reported to improve symptoms significantly sooner than PPI [27]. Notably, heartburn was completely relieved in 31.3% of patients on Day 1 with VPZ. In mild RE, the goal of treatment should be to relieve symptoms rather than mucosal healing. Step-down therapy might be reasonable for mild RE, assuming that VPZ 20 mg for 4 weeks is not always necessary for mild RE.

The present study had some limitations. First, it was a retrospective study with a small sample size. Second, this was a single-arm study without a control group. Third, endoscopic evaluation after step-down therapy was lacking. Fourth, we used FSSG for the inclusion criteria and evaluation of efficacy, which could introduce a bias (a regression to mean). Finally, long-term observation of treatment efficacy was also lacking. Despite these limitations, to our knowledge, this is the first study to focus on PRO and report the efficacy of tapering therapy using VPZ for symptomatic patients with mild RE. We also believe that this new treatment strategy can be an option in maintenance period aiming to optimize drug administration for each patient in mild RE. Further studies using multivariate analysis will be needed to elucidate the attributes of patients who benefit most from step-down therapy using VPZ. Moreover, a prospective study is required to verify the efficacy of step-down therapy using VPZ.

## 5. Conclusions

In conclusion, step-down therapy using VPZ may be a viable treatment option for symptomatic patients with mild RE. Notably, step-down therapy could improve the reduction of VPZ intake compared with treatments based on current guidelines. We believe that the treatment of GERD with mild RE should be based on symptoms, thereby avoiding unnecessary long-term administration of acid suppressants. Further studies with larger numbers of patients are required, and PRO of GERD treatment should be the focus of future research.

## Figures and Tables

**Table 1:** Degree of disability of daily life due to discomfort of GERD (*D* score).

**Score**	**Definition**
0	GERD symptoms do not interfere with daily life
1	GERD symptoms mildly interfere with daily life
2	GERD symptoms moderately interfere with daily life
3	GERD symptoms severely interfere with daily life

Abbreviation: GERD, gastroesophageal reflux disease.

**Table 2:** Patient characteristics.

	**N** = 63
Age, median (years)	70 (44–86)
Sex, male (%)	40 (63.5)
Height, median (cm)	163.4 (147.3–177.1)
Weight, median (kg)	61.7 (32–86.5)
Body mass index (kg/m^2^), median	23.5 (14.2–30.3)
Los Angeles classification (A : B), *n*	37 : 26
PPI use before step-down therapy, *n* (%)	21 (33.3)

Abbreviation: PPI, proton pump inhibitor.

**Table 3:** Primary endpoint and other outcomes.

	**N** = 63
Decrease of FSSG by ≥ 3 points, *n* (%)	48 (76.2)
Decrease of FSSG by ≥ 1 point, *n* (%)	56 (88.9)
Decrease of FSSG to ≤ 7 points, *n* (%)	33 (52.4)
FSSG score before step-down therapy, median (points)	13 (8–35)⁣^∗^
FSSG score after step-down therapy, median (points)	7 (0–29)⁣^∗^
FSSG heartburn score before step-down therapy, median (points)	2 (0–4)⁣^∗^
FSSG heartburn score after step-down therapy, median (points)	1 (0–3)⁣^∗^

Abbreviations: FSSG, frequency scale for the symptoms of gastroesophageal reflux disease; VPZ, vonoprazan.

⁣^∗^Statistical significance of *p* < 0.01.

**Table 4:** Step-down therapy using VPZ and the median duration of VPZ intake during 28 days.

	**N** = 63
Patients who stepped down VPZ, *n* (%)	45 (71.4)
Patients who could not step down VPZ, *n* (%)	18 (28.6)
Patients who discontinued VPZ, *n* (%)	29 (46)
Patients who restarted VPZ, *n* (%)	21 (72.4)
Duration of 20 mg VPZ intake, median (days)	11 (1–28)
Duration of 10 mg VPZ intake, median (days)	3 (0–26)

Abbreviation: VPZ, vonoprazan.

**Table 5:** Subgroup analysis.

	**Before step-down therapy**	**After step-down therapy**
FSSG score with LA Grade A (*n* = 37), median (points)	13 (8–35)	8 (0–29)⁣^∗^
FSSG score with LA Grade B (*n* = 26), median (points)	12 (8–26)	6 (0–18)⁣^∗^
FSSG score of PPI-naïve patients (*n* = 42), median (points)	12.5 (8–35)	7 (0–29)⁣^∗^
FSSG score of PPI users (*n* = 21), median (points)	13 (9–26)	7 (1–23)^†^

Abbreviations: FSSG, frequency scale for symptoms of gastroesophageal reflux disease; PPI, proton pump inhibitor.

⁣^∗^Statistical significance of *p* < 0.01.

^†^Statistical significance of *p* = 0.01.

## Data Availability

The data used to support the findings of this study are available from the corresponding author upon reasonable request.
